# Exploring the Product Owner Role Within SAFe Implementation in a Multinational Enterprise

**DOI:** 10.1007/978-3-030-58858-8_10

**Published:** 2020-08-18

**Authors:** Daniel Remta, Michal Doležel, Alena Buchalcevová

**Affiliations:** 6 IT University of Copenhagen, Copenhagen, Denmark; 7 University of British Columbia, Vancouver, BC Canada; University of Economics, Prague, Czech Republic

**Keywords:** Product Owner, Responsibilities, Functions, Scaled Agile Framework, SAFe, Large-scale agile methods

## Abstract

[Context] Agile development methods are highly popular across software organizations. To leverage benefits in larger enterprises, Agile development methods have to be scaled. Scaled Agile Framework (SAFe) is the most commonly used scaling framework. Performing of the Product Owner role has been identified as crucial in project success in large-scale environments. Staffing the right Product Owner is one of the challenges of adopting SAFe. [Motivation] Research papers focused on Product Owner in SAFe are scarce. Our study outcomes help enterprises to understand the Product Owner role in SAFe and therefore contribute to the removal of challenges with finding the right Product Owners. Additionally, we aim to improve the research community’s understanding of the Product Owner role within the context of SAFe. [Method] Qualitative data were collected through three semi-structured interviews and analyzed using deductive content analysis. [Results] This paper presents the initial results of a single case study. We found out that many activities identified for Product Owners in previous research are not carried out by Product Owners in this particular SAFe implementation.

## Introduction

Agile, as a set of iterative and incremental software engineering methods [1], becomes commonplace in many large organizations [2], where agile practices have to be scaled [3]. Scaling Agile was identified as an important research topic [4], and more and more large organizations are transitioning towards Agile [1, 2]. During this process, new roles are introduced to the enterprise environment, such as the Product Owner (PO) role. The PO role originates from the Scrum method [5] and has been identified to play a crucial role in project success [1]. The PO role is also present in the frameworks for large-scale Agile, such as Scaled Agile Framework (SAFe) [6], which is the most commonly used framework for scaling Agile [7]. However, staffing the right PO was identified as one of the challenges of adopting SAFe [8]. Surprisingly, research papers with a focus on the PO role in SAFe are scarce. A better understanding of the role in SAFe will help enterprises to acknowledge requirements to people assigned to the PO role and therefore help with the success of their SAFe adoption. In our research, we address the following research question (RQ): Which of the activities described for Product Owners in previous research are performed by the Product Owners in the examined enterprise that follows SAFe?

The paper is organized as follows. Following Introduction, Sect. 2 provides a literature review and a taxonomy of PO functions mapped to SAFe roles. In Sect. 3, the adopted research method and the context of the study are described. Section 4 provides findings from the semi-structured interviews. Section 5 concludes the paper with a discussion and a summary of key take-aways.

## Background

In this section, we provide a literature review and present a taxonomy of PO functions. We use the term “function” to refer to a designated group of coupled activities.

### Large-Scale Agile.

Scaling Agile was identified as an important topic [4]. Dikert et al. [1] identified challenges and success factors for large-scale Agile transformations, focusing on large-scale organizations with 50 or more people. The agile practices have to be scaled [3], and POs must cope with a range of new activities [9].

### Product Owner.

Originally, Scrum defined the PO role relatively simply as the sole person responsible for managing the Product Backlog [5]. Yet, the adequate performing of Product Owners was identified as one of the critical success factors for projects [1], and a wide range of other activities performed by PO was described [3, 10–14]. The PO role has been examined in previous research. Bertzen and Moe  [15] identified the importance of frequent communication and interaction between POs in large-scale Agile. Paasivaara et al. [3] described the differences in approaches to scaling the PO role. Bass carried out an empirical research [9, 10] on the PO activities in a large-scale Agile environment. Yet, only a limited amount of papers focus on the PO role in SAFe.

### SAFe.

SAFe provides prescriptive guidelines for implementing enterprise-scale Lean-Agile development, and a number of companies that have applied SAFe have reported significant benefits from it [6]. Interestingly, the popularity of SAFe seems unaffected by concerns about Agile principles and values in the top-down approach, and SAFe’s strong emphasis on process rather than on people [16, 17].

### SAFe and PO.

Despite some existing research on SAFe  [8], papers focused on the PO role in SAFe are still scarce. The study of Paasivaara et al. [2] states that implementing SAFe results in closer collaboration and communication between POs and Product Managers (PM). However, the study focus was on SAFe adoption in general. Overall, little is known about the PO in SAFe.

### PO in SAFe.

SAFe adopted the PO role but, in contrast to Scrum, assumes that a single person cannot handle product and market strategy while also being dedicated to agile teams. It was confirmed in [9, 10] that in large-scale Agile environments, the scope of activities goes beyond the capacity of one person acting as PO. PO and PM share responsibilities for working with customers [6]. We have identified some of the PO activities from [3, 10–14] in the descriptions of activities performed by other SAFe roles. The System Architect (SA) creates an architectural vision and aligns teams around a shared technical direction [6]. Aside from development, during planning, the Agile Team (AT) identifies risks and impediments [6]. The risks are actively managed by the Release Train Engineer (RTE) [6]. Specialty roles, people, and services to cover customer training, support, and compliance audits are represented in Shared Services [6]. Scrum Master (SM) ensures that the agile processes and guidelines are followed [6].

### Taxonomy of PO Functions.

To understand the fragmentation of PO activities, we extracted the taxonomy of the PO functions from the previous research on POs outside the context of SAFe [3, 10–14]. Next, we mapped the activities from the taxonomy to the descriptions of roles provided in SAFe [6]. The results are presented in Table 1. The functions described by Bass [10, 11] were used in accordance with his descriptions. When adding functions from [3, 12–14], we followed the example of the descriptions of functions in [10, 11]. Added functions from [3, 12–14] are written in italics.

**Table 1.** PO functions taxonomy mapped to SAFe. (Synthetized from [3, 6, 10–14].)

Function	Activity	Role in SAFe
Prioritizer [10]	Prioritizes requirements in the product or sprint backlog and ensures requirements bring value to the business.	PO/PM
Groom [10]	Makes sure product backlog is continuously evolving and registers requirements from clients. Clarifies the details of product backlog items and their respective acceptance criteria.	PO/PM
Release Master [10]	Manages and approves release schedules.	PM
Technical Architect [10]	Designs, implements, and disseminates a reference architecture, provides architecture coordination on large projects.	SA
Governor [10]	Provides a technical governance framework to project teams working on a program and ensures project compliance with corporate guidelines and policies.	RTE/SM/SA
Communicator [10]	Ensures that global teams are in sync and bridges onshore and offshore geographical distribution.	SM/PO
Traveler [10]	Travels to clients to get first-hand knowledge of the client’s needs and gathers an understanding of it by spending time onshore at customer sites.	PM/PO
Intermediary [10]	Has extensive experience in the system business domain and acts as an interface to senior executives.	PM
Risk Assessor [10]	Performs risk management and mitigation when needed, especially with focus on technical complexity.	AT/RTE
Gatekeeper [11]	Determines feature or story completeness for inclusion in the release.	PO
Customer Relationship Manager [11]	Provides technical support to customers, assists with site preparation and product installation, and conducts product training.	Shared Services
*Entrepreneur* [12]	*Develops business/product plan and service planning.*	PM
*Motivator* [13]	*Motivates the team and finds ways to get the team more involved.*	SM
*Leader* [14]	*Leads the development teams.*	SM
*Negotiator* [3]	*Negotiates with different stakeholders to avoid conflicts.*	PM/PO

The mapping in Table 1 indicates the fundamental difference between the PO role in SAFe and the PO role examined in previous research by showing a visible fragmentation of previously identified PO activities to other SAFe roles.

## Research Method

### Context.

The single-case study has been conducted in a division of multinational enterprise that delivers mainframe software. The examined value stream specifically focuses on workload automation software, written mostly in low-level programming languages. For newer components (e.g. modern user interfaces), higher languages like C, Java, or JavaScript are used. The division follows the Portfolio SAFe configuration [6] and claims to follow SAFe on the team and program level “by the book”. The development teams work in 2- to 4-week sprints. Overall, the environment can be characterized as a very large-scale Agile with 30 teams and 250+ team members.

### Data Collection.

Three POs have participated in the initial phase of the study. Table 2 shows the length of their experience in the role, the number of teams they concurrently work with, the number of development team members they work with, their previous position, and overall experience in the field of mainframe enterprise software. Additional interviews with 6–7 respondents are planned to complete the study.

**Table 2.** Research participants

PO	Months in the role	Teams	Members	Previous position	Years in field
PO_A_	14	3	16	Developer, Scrum Master	3
PO_B_	12	2	16	Developer	5
PO_C_	10	1	16	Software support engineer	22

The interviews were done in person, audio-recorded, and transcribed. The interview guide is available at https://rebrand.ly/4ylvsbo. The interviews consisted of 54 questions, 3 to 4 per each function, and took from 65 to 85 min to complete. Our aim was to identify if the activity of the function had been performed, to what extent, how often, typically followed by a request to provide an example to validate the previous answers. All recordings and transcripts were given codes and stored separately from any names or other direct identification of participants.

### Data Analysis.

A deductive content analysis [18] was used. We used the functions from the taxonomy introduced in Table 1 as categories for the analysis. Next, we manually open coded, analyzed, and matched data into these categories. As the last step, we have interpreted the results.

## Results

In this section, we provide the preliminary results from the examined SAFe implementation. The comparison of each PO function from the taxonomy introduced in Table 1 with the findings from the semi-structured interviews showed that POs in the selected enterprise SAFe implementation perform the activities of Prioritizer, Groom, Communicator, Traveler, Gatekeeper, and Motivator. In the rest of the functions, POs are only partially involved. In Table 3, we present evidence for each function in the form of quotations or merged findings. The functions confirmed as being performed by POs are written in bold.

**Table 3.** PO Functions in SAFe – findings. Source: Interviews

Function	Commentary
**Prioritizer**	*“PI objectives are our highest priority […] if we have some customer interest in the feature or the story, it will get prioritized higher. I try to keep my thoughts out of it. I try to prioritize first based on customer, then on business needs.” [PO*_*C*_*]*
**Groom**	*“[…] continuously going through the backlog, seeing if there are stories to be delivered and updated, as we are learning about new technologies and based on the feedback from the customer I go through the stories and change them or update them, and move into the direction we want to go.” [PO*_*C*_*]*
Release master	PO’s main activity in the release process is ensuring completion of the release checklist, which has to be signed off by involved stakeholders representing different business units. PO_A_ described PM as the only one responsible. *“Release dates, numbering, names, all of this stuff goes to the product manager, and it is his responsibility.” [PO*_*A*_*]*
Technical architect	All POs stated that architectural activities and decisions are not in the scope of PO activities. As evident from PO_C_’s answer, they try to avoid any architectural involvement as much as possible. “*I try very hard to exclude myself from any involvement with architectural decisions. And I also try very hard to avoid anything in the description of the user stories that will imply any suggestion for architectural decision.” [PO*_*C*_*]*
Governor	Different roles perform governance activities. POs mentioned: SA, Legal, Functional Managers, and SMs. For POs, the delivery of the item is the primary concern. POs only make sure that the frameworks are followed and, in case not, inform other roles to take action. “*I raise a point or question when I believe it is appropriate to raise the point, but I don’t see myself as a policeman being on guard, or anything [like that].” [PO*_*B*_*]*
**Communicator**	*“I have team members in Texas, in Pittsburg, North Virginia, and Sydney, and it is very difficult to manage the team across so many different time zones.” [PO*_*C*_*]*
**Traveler**	*“[…] mostly technical conferences, and this year I want to continue in US and Europe. I am also expecting to do a few customer visits*. *[PO*_*C*_*]”*
Intermediary	All POs felt experienced in the domain of enterprise software development, but PO_B_ and PO_C_ were not that confident in the domain of Product Ownership. POs confirmed communications with senior executives; however, as the senior executives, they understand PMs, customer Account Managers, and Business Owners. “[…] *in general PM and upper product management, while I talk most of the time with PM*.” *[PO*_*B*_*]*
Risk assessor	All POs do identify and articulate potential risks but do not participate in risk mitigation. The mitigation is understood as belonging to other roles. *“Other people will go and take care of this. It is not a product owner’s responsibility to solve it; it is a PO’s responsibility [just] to raise it.” [PO*_*A*_*]*
**Gatekeeper**	*“I consider it complete when engineers will demo it, will show to me and the other stakeholders what they did, elect feedback, and I don’t consider it complete when there will not be proper QA [Quality Assurance] testing on it to make sure what we have is working in that front.” [PO*_*A*_*]*
Customer relationship manager	All POs clearly stated that there are dedicated support and education teams, so POs are not directly involved in providing support nor training to the customers. PO activities in this area are limited to providing internal training sessions and materials to support and education teams. “*I got the skillset, but it is not what I do. I will not sit with the client and show him how to install the product.*” *[PO*_*B*_*]*
Entrepreneur	All POs are involved and are contributing by providing information to, and having discussions with, PM. The decisions and plans are made by PM, who also owns the product roadmap. *“As PO I just discuss what customer want, reflect this to the management, and then the decisions will come from the management, not the product owner.” [PO*_*A*_*]*
**Motivator**	All POs stated motivating the team, and people is part of their job. “[…] *give KUDOS to people who deserve it, always pull out someone and say ‘Hey, good job!’, in case they did a good job. If someone did an outstanding job, you should communicate this to their managers and directors*. *[PO*_*A*_*]*
Leader	None of the POs identified himself as the leader of the team. *“No. I don’t consider myself being a leader to the team. But I don’t even know who the leader for the team is.” [PO*_*B*_*]*
Negotiator	POs seem to be positioned as mediators, but not the negotiators *per se*. *“I would consider myself a mediator between different stakeholders and trying to find the source of the conflict and initiator of it.” [PO*_*C*_*]*

## Discussion and Summary

Examining the Scaled Agile Framework [6] and its concrete implementation in a multinational enterprise, we addressed the RQ: *“*Which of the activities described for Product Owners in previous research are performed by the Product Owners in the examined enterprise using SAFe?”. We found out that many activities identified for PO in previous research are not carried out by POs in this SAFe implementation. Specifically, our preliminary research findings demonstrated that PO in this particular SAFe implementation is *not*: leading the teams; mitigating identified risks; designing, implementing and disseminating technical architecture; providing governance frameworks; ensuring compliance with corporate guidelines; communicating with senior executives; nor providing support and customer training. This is surprising because previous research conducted outside the context of SAFe assumed that PO responsibilities are very broad. Below, we offer a hypothesis about the reason for the inconsistency.

The mapping provided in Table 1 indicated that there is a complex relationship between the previously identified PO functions and roles in SAFe, where the performing of the activities is fragmented among multiple roles. Supported by the outcomes from the conducted semi-structured interviews, which indicate that in SAFe, the original PO role functions have undergone significant modifications, it is evident that PO functions in SAFe are different from the functions of the PO role identified in previous research. In our research, the close collaboration between PO and PM described in [2] was confirmed. In fact, PO’s decisions about priorities and requirements for development are strongly influenced by PM, who, in reality, drives and develops business and product plans. This PM activity contradicts the prime Scrum definition of PO as the sole person responsible for managing the Product Backlog [5]. Therefore, the PO’s accountability for product leadership fades away.

We offer a possible explanation. SAFe provides rigorous descriptions for the implementation of Agile processes on a large-scale and put the emphasis on the process [15]. This *de-facto* leads to a new form of the management-driven top-down approach [16] with the fragmentation of the roles. We support this statement by evidence from the interview: *“As PO I just discuss what customer want, reflect this to the management, and then the decisions will come from the management, not the product owner.” [PO*_*A*_*]*

### Conclusion.

The PO role in the examined SAFe implementation deviates from the previous understanding of the role outside the context of SAFe, as the range of PO activities is narrowed. We attribute it to the top-down approach criticized by practitioners and confirmed during our single-case study. We identified PM as the person accountable for, and steering, the product.

### Limitations and Threats to Validity.

The presented results were obtained in one division of a large multinational enterprise and, therefore, might be influenced by the local context. Achieving a validity in a single case study is a known challenge, thus plausible, rival explanations or triangulation methods should be used in further research to strengthen validity of our findings [19]. The study provides only preliminary outcomes from the three interviews. Hence, it is too soon generalize the PO role in SAFe. Two papers [12, 13] used in our taxonomy did not explore a large-scale environment context. Still, we identified similar activities appearing in the examined environment.

### Future Works.

As this research is ongoing, another 6–7 interviews will follow to validate the preliminary findings. Our next steps will be to gather, analyze, and incorporate more data from POs with different experience and from different parts of the organization, which will help to understand the real-life PO functions in SAFe implementations. More case studies exploring further enterprise SAFe adoptions are needed to confirm the findings presented in this study.

## References

[1] Dikert K, Paasivaara M, Lassenius C (2016). Challenges and success factors for large-scale agile transformations: a systematic literature review. J. Syst. Softw..

[2] Paasivaara, M.: Adopting SAFe to scale agile in a globally distributed organization. In: ICGSE, pp. 36–40 (2017)

[3] Paasivaara, M., Heikkila, V.T., Lassenius, C.: Experiences in scaling the product owner role in large-scale globally distributed scrum. In: ICGSE, pp. 174–178 (2012)

[4] Dingsøyr T, Moe NB (2013). Research challenges in large-scale agile software development. SIGSOFT Softw. Eng. Notes.

[5] Schwaber, K., Sutherland, J.: The Definitive Guide to Scrum: The Rules of the Game (2017). https://www.scrumguides.org/docs/scrumguide/v2017/2017-Scrum-Guide-US.pdf

[6] SAFe: Scaled agile framework (2019). http://www.scaledagileframework.com/

[7] CollabNet VersionOne: The 13th State of Agile Survey (2019). https://www.stateofagile.com/

[8] Putta, A., Paasivaara, M., Lassenius, C.: Adopting scaled agile framework (SAFe): a multivocal literature review. In: XP’18, pp. 39:1–39:4 (2018)

[9] Bass JM, Haxby A (2019). Tailoring product ownership in large-scale agile projects: managing scale, distance, and governance. IEEE Softw..

[10] Bass JM (2015). How product owner teams scale agile methods to large distributed enterprises. Empir. Softw. Eng..

[11] Bass, J., Beecham, S., Razzak, M.A., et al.: Poster: an empirical study of the product owner role in scrum. In: ICSE, pp. 123–124 (2018)

[12] Oomen, S., Waal, B.D., Albertin, A., Ravesteyn, P.: How can Scrum be succesful? Competences of the scrum product owner. In: ECIS, pp. 130–142 (2017)

[13] Raithatha, D.: Making the whole product agile – a product owners perspective. In: XP, pp. 184–187 (2007)

[14] Kristinsdottir, S., Larusdottir, M., Cajander, Å.: Responsibilities and challenges of product owners at spotify - an exploratory case study. In: HCSE/HESSD, pp. 3–16 (2016)

[15] Berntzen, M., Moe, N.B., Stray, V.: The product owner in large-scale agile: an empirical study through the lens of relational coordination theory. In: XP, pp. 121–136 (2019)

[16] Jeffries, R.: Issues with SAFe (2014). https://ronjeffries.com/xprog/articles/issues-with-safe/. Accessed 22 Mar 2020

[17] Elssamadisy, A.: Has SAFe Cracked the Large Agile Adoption Nut? (2013). https://www.infoq.com/news/2013/08/safe/#. Accessed 22 Mar 2020

[18] DeFranco JF, Laplante PA (2017). A content analysis process for qualitative software engineering research. Innov. Syst. Softw. Eng..

[19] Yin RK (2013). Validity and generalization in future case study evaluations. Evaluation.

